# Upregulation of cathepsin D in the caudate nucleus of primates with experimental parkinsonism

**DOI:** 10.1186/1750-1326-6-52

**Published:** 2011-07-21

**Authors:** Sowmya V Yelamanchili, Amrita Datta Chaudhuri, Claudia T Flynn, Howard S Fox

**Affiliations:** 1 Department of Pharmacology and Experimental Neuroscience, University of Nebraska Medical Center, Omaha, NE 68198, USA; 2 Department of Immunology and Microbial Science, The Scripps Research Institute, La Jolla, CA 92037, USA; 3 Molecular and Integrative Neuroscience Department, The Scripps Research Institute, La Jolla, CA 92037, USA

**Keywords:** Parkinson's, MPTP, striatum, caudate, neurodegeneration, cathepsin, apoptosis, nonhuman primate

## Abstract

**Background:**

In Parkinson's disease there is progressive loss of dopamine containing neurons in the substantia nigra pars compacta. The neuronal damage is not limited to the substantia nigra but progresses to other regions of brain, leading to loss of motor control as well as cognitive abnormalities. The purpose of this study was to examine causes of progressive damage in the caudate nucleus, which plays a major role in motor coordination and cognition, in experimental Parkinson's disease.

**Results:**

Using chronic 1-methyl-4phenyl-1,2,3,6-tetrahydropyridine treatment of rhesus monkeys to model Parkinson's disease, we found a upregulation of Cathepsin D, a lysosomal aspartic protease, in the caudate nucleus of treated monkeys. Immunofluorescence analysis of caudate nucleus brain tissue showed that the number of lysosomes increased concurrently with the increase in Cathepsin D in neurons. *In vitro *overexpression of Cathepsin D in a human neuroblastoma cell line led to a significant increase in the number of the lysosomes. Such expression also resulted in extralysosomal Cathepsin D and was accompanied by significant neuronal death associated with caspase activation. We examined apoptotic markers and found a strong correlation of Cathepsin D overexpression to apoptosis.

**Conclusions:**

Following damage to the substantia nigra resulting in experimental Parkinson's disease, we have identified pathological changes in the caudate nucleus, a likely site of changes leading to the progression of disease. Cathepsin D, implicated in pathogenic mechanisms in other disorders, was increased, and our *in vitro *studies revealed its overexpression leads to cellular damage and death. This work provides important clues to the progression of Parkinson's, and provides a new target for strategies to ameliorate the progression of this disease.

## Background

Parkinson's disease (PD) is the second most common neurodegenerative disease. Apart from its genetic predisposition, most cases arise sporadically and factors including drugs and toxic chemicals have been demonstrated to induce PD [1]. While medical (e.g. L-3,4-dihydroxypheylalanine, L-DOPA) and surgical (e.g. deep brain stimulation) therapies have been effective in the treatment of PD to a certain extent, nothing to date is capable of arresting disease progression.

In PD, there is progressive loss of dopamine (DA) containing neurons in the substantia nigra (SN) pars compacta. Motor symptoms initially dominate the clinical picture, and as the disease progresses cognitive abnormalities are often evident [1,2]. While numerous studies examine damage and neuronal loss in the SN, other neuronal systems and brain regions are also affected [3]; and the nature of this additional neuronal damage is relatively unknown.

Understanding the dysregulation of genes and proteins involved in neuronal dysfunction and disease progression can open avenues for new therapeutic targets in PD. One main outflow tract from the SN is to the striatum where the presynaptic dopaminergic terminals originating in the SN are lost [4]. To address other PD-related changes in the striatum, we targeted the caudate nucleus (CN), a part of the striatum that not only receives dopaminergic input from the SN as well as the ventral tegmental area but also glutaminergic projections from prefrontal associational and anterior cingulate limbic areas. Similar to other regions of the striatum, the CN is involved in motor function and also has an important role in cognition. Imaging studies have linked abnormalities in the striatum to altered cognitive executive functions in those with PD [5-9].

A common cord in many aspects of neuronal survival is the lysosomal pathways [10]. The lysosomal proteases, which play a key role in intracellular proteolysis and in extracellular remodeling [11], are important in maintaining homeostasis by exerting degradation and regulatory functions. Lysosomal dysfunctions have been specifically associated with degenerative phenomenon [12] as well as with age-related neurological diseases [13]. Among the most powerful hydrolytic enzymes in the lysosomes are the cathepsins. Cathepsin D (Cat D) is the major intracellular aspartic protease and is present in relatively high concentrations within the lysosomes. Previous studies in a variety of cell systems such as endothelial cells, T-lymphocytes and fibroblasts have shown that increases in Cat D leads to apoptosis (reviewed in [14]). Cat D upregulation has been associated with neurodegenerative disorders including Alzheimer's disease (AD) and its presence extracellularly in senile plaques was clearly noted [15]. The upregulation of Cat D was shown to occur at an early stage in experimental models of AD leading to slow apoptosis of neurons [16]. Therefore, we questioned whether deregulation of lysosomal Cat D is involved in the progressive neuronal damage as seen in PD, focusing on the CN.

We hypothesized that Cat D can act as a molecular trigger for neuronal damage in CN. Using a chronic 1-methyl-4phenyl-1,2,3,6-tetrahydropyridine (MPTP) treated rhesus monkey model, we assessed the expression level of Cat D in CN followed by immunohistochemical analysis on brain sections. Next, we performed *in vitro *overexpression of Cat D and subsequently followed the results by functional and imaging studies to confirm our hypothesis.

## Results

### MPTP-treatment of monkeys

Monkeys are sensitive to the PD-like effects of MPTP and have a good correspondence of brain function, neurochemistry, and neuroanatomy to humans. In monkeys, both acute and chronic dosing protocols can lead to a PD-like disease. While the acute effects of high dose MPTP in experimental animals can be dramatic, the acute toxicity differs from the course of PD. In addition, functional recovery can occur in a variety of dosing protocols.

Chronic MPTP dosing cannot only lead to stable motor deficits but also cognitive abnormalities as seen in PD. In order to better mimic the chronic nature of PD, we utilized a repeated low-dose protocol, in which animals received a dose of 0.3-0.4 mg/kg MPTP on two consecutive days, followed by assessment of stable effects of the treatment at 3-4 weeks post-treatment. Animals then received repeat dosing in order to achieve a state of stable, mild functional deficits. Following treatment, animals displayed behavioral changes (less grooming and social interaction when housed with another monkey and aggressiveness to humans) and spent more time lying down. Some monkeys showed a dramatically impaired performance on a bimanual motor skills (BMS) task, with slower times to perform the test or no interest at all.

**Figure 1:**
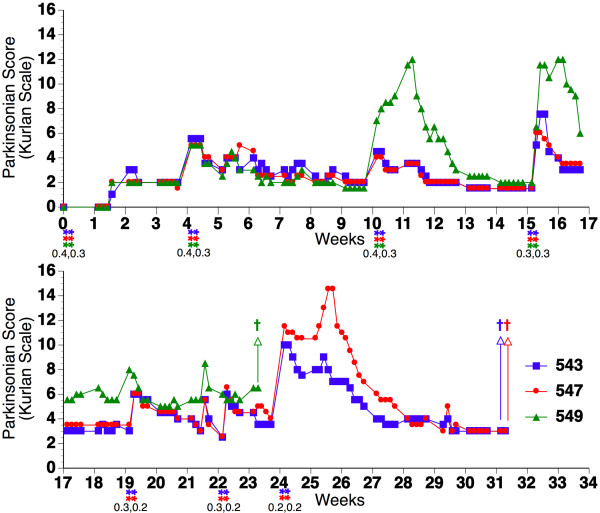
**MPTP treatments and clinical score of monkeys**. The rhesus monkeys were treated with MPTP at the time points indicated by the color-coded asterisks. Doses were given on two consecutive days at the amounts (in mg/kg) indicated under the asterisks. The Kurlan rating score (rating the animal's posture, gait, tremor, general mobility, hand movements, climbing, holding food, eating, balance, gross motor skills, and defense reaction) is given for each monkey over time. Untreated control animals and animals before MPTP treatment had values of 0 (normal), higher values indicate increased deficiency. Daggers indicate time of sacrifice of each animal.

**Figure 2:**
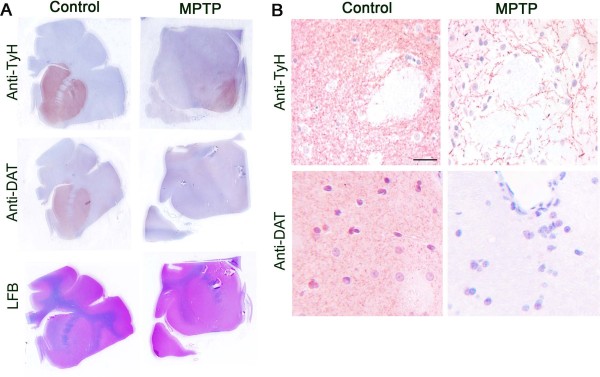
**Loss of dopaminamergic nerve terminal markers in the striatum of MPTP treated monkeys**. (A) Immunohistochemical staining reveals strong staining of striatal sections of control brains (left panel: top and middle) for tyrosine hydroxylase and dopamine transporter; whereas, MPTP administered monkey striatal sections (right panel: top and middle) show a minimal staining. The bottom panels are sections stained with luxol fast blue. The images are unmagnified scans of the slides. (B) Immunohistochemical staining reveals strong staining of striatal sections a control brains (left) for TyH and DAT; whereas, in an MPTP administered monkey, striatal sections (right) show minimal staining, Bar = 20 μm. The images are representative of the monkeys in each group.

Signs of PD-like disease were scored using the Kurlan scale [17], considered to be the optimal clinical rating scale for MPTP-induced Parkinsonism in macaques [18]. Through this dosing and rating system, we induced a state of mild stable Parkinsonism (Kurlan scores of 3-6.5 persisting a minimum of 7 weeks following the last dose, Table 1, Figure 1). Histopathological analysis of the dorsal striatum clearly showed a profound decrease in tyrosine hydroxylase (TyH) [Figure 2A (upper panel), 2B (upper panel)] as well as dopamine transporter (DAT) [Figure 2A (middle panel), 2B (lower panel)] staining in the MPTP-treated monkeys compared to untreated controls, revealing persistent damage to the pre-synaptic dopaminergic terminals from the SN.

**Table 1:** MPTP-treated animal subjects.

*Animal*	*Total MPTP (mg/kg)*	*Weeks since first treatment*	*Weeks since last dose*	*Final Kurlan score*
543	4.1	31	7	3
547	4.1	31	7	3
549	2.7	23	8	6.5

Animals received intermittent low-dose MPTP as indicated in Figure 1. The total dose of MPTP received by each animal, total time since first treatment, the time between the last dose and animal sacrifice, and the clinical score at sacrifice is indicated.

### Upregulation of Cat D mRNA and protein in MPTP-treated monkey CN

**Figure 3:**
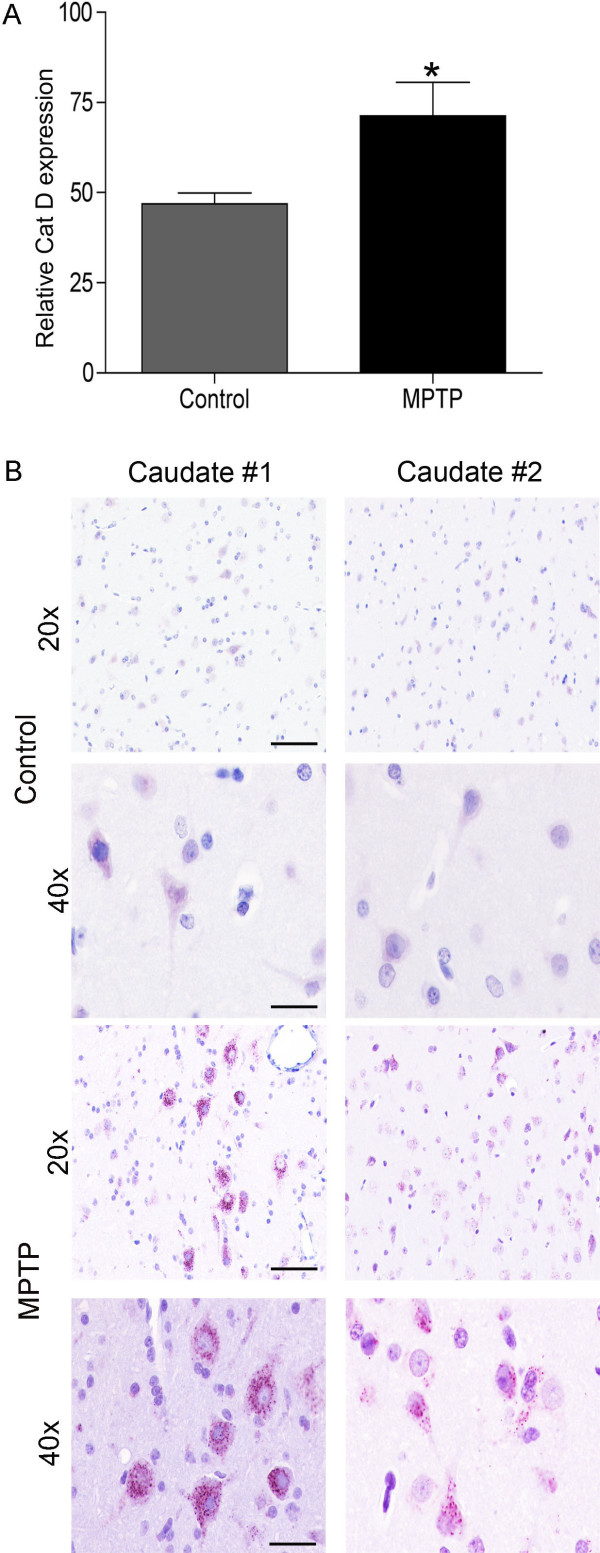
**Cat D is upregulated in MPTP caudate nucleus**. (A) Taqman qRTPCR for Cat D mRNA was performed on RNA from caudate samples. A significant upregulation in Cat D mRNA is seen. The delta Ct (dCt) method was performed to determine relative concentrations, using the average of the Ct of 18S and GAPDH as the normalizing value. Mean and standard error of the mean shown, significance is indicated by *p < 0.05 (Student's t-test). (B) Photomicrographs of representative sections of caudate of two animals (#1, #2) from each group of the control and MPTP monkeys. Caudate sections were immunohistochemically stained with anti-Cat D. Minimal staining is seen in sections from control animals (bar = 50 μm) and in corresponding increased magnification (40×, bar = 10 μm); whereas, in MPTP treated animal, there is increased staining. Increased magnification (40×, bar = 10 μm) reveals that the staining is seen within neurons in the cell body, axon hillock (bottom right).

In order to assess whether neurodegenerative mechanisms were ongoing in the CN long after the termination of MPTP treatment, we assessed the expression of Cat D. We first analyzed if Cat D is altered at the mRNA level. We extracted RNA from CN of the MPTP treated monkeys and quantified Cat D mRNA by quantitative real-time PCR in comparison to caudate RNA from four untreated control monkeys. We found a significant increase (p < 0.05) in Cat D mRNA levels in chronically MPTP-treated animals (Figure 3A).

**Figure 4:**
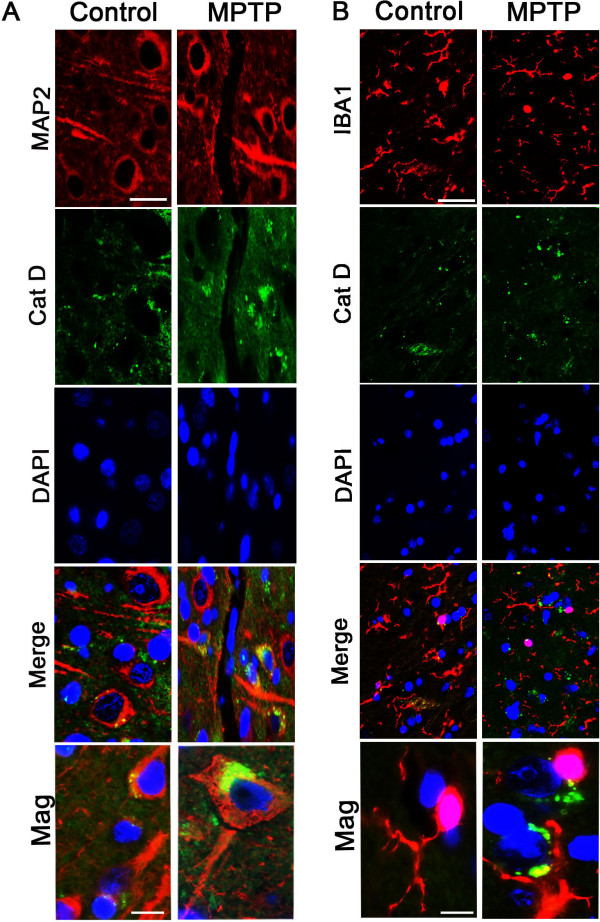
**Double immunofluorescence of CatD with MAP2 and IBA1 in CN od MPTP monkey**. (A) Double immunostaining was performed on MPTP CN sections with the neuronal marker anti-MAP2 (red) and anti-Cat D (green). The merged image illustrates a co-localization of Cat D with MAP2, confirming its presence in neurons. Bar = 20 μm. Higher magnification of single neuron is also provided (bottom panel, Mag). Bar = 5 μm. (B) Double immunostaining was performed on MPTP CN sections with the microglial marker anti-IBA1 (red) and anti-Cat D (green). The merged staining of Cat D with IBA1 conforms the absence of Cat D in microglial cells. Bar = 20 μm. Higher magnification (bottom panel, Mag). Bar = 5 μm.

Next, we investigated which cell types in CN show increased production of Cat D protein expression. As seen in Figure 3B, there is increased Cat D immunoreactivity in the neurons of MPTP-treated monkey CN. Interestingly, there is increased staining in soma and axonal cones. Intriguingly, this staining pattern has been previously reported to be present in AD [15]. Furthermore, we examined co-localization of Cat D and the neuronal marker MAP2 and ascertained that its increased expression is indeed in neurons (Figure 4A). Additionally, we examined if Cat D co-localizes with IBA1, a microglial marker; however we did not observe distinct expression of Cat D in microglial cells (Figure 4B).

**Figure 5:**
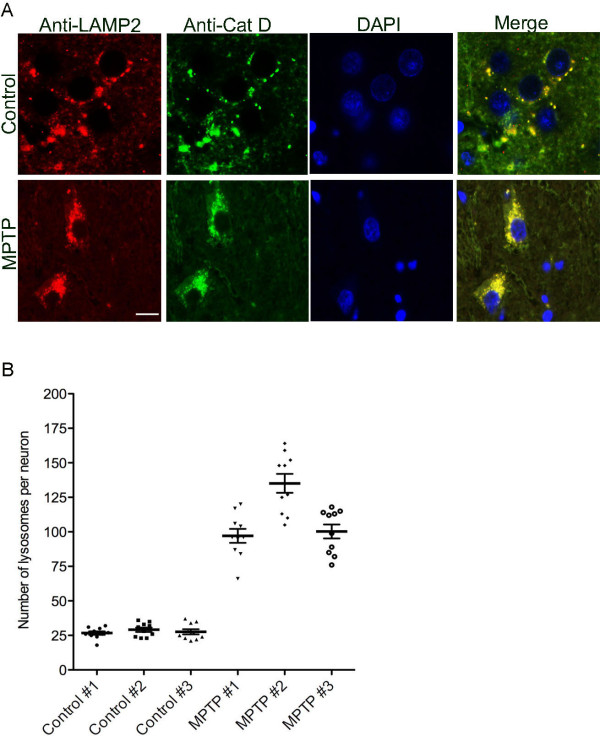
**Lysosomal localization of Cat D**. (A) Double immunostaining was performed on control and MPTP CN sections with anti-Cat D (green) and the lysosomal marker anti-LAMP2 (red). The merged image (right panel) illustrates a co-localization of Cat D with LAMP2, its increase in sections from MPTP treated animals. Bar = 20 μm. (B) Quantification of lysosomes was performed on sections, n = 10 neurons for each animal were examined, unpaired t-test was performed on the average lysosomes quantified from the three control (Mean ± SEM: 27.8 ± 0.89) and three MPTP (Mean ± SEM: 110.8 ± 4.5) animals, p < 0.001.

Proper regulation of cathepsin levels is important in neurons, and upregulation as well as absence of cathepsins have considerable consequences on the maintenance and function of nervous system. Lysosomal proteases are rarely secreted outside the cells under normal conditions. Normal levels of Cat D have the ability to digest > 95% of total brain protein within 24 hr under *in vitro *conditions [19], implicating that the leakage of lysosomal compartments can have profound deleterious effects on the cell itself and if released outside the cells would effect the surrounding brain milieu. To investigate the relationship of Cat D to lysosomes in the brains, we performed double immunofluorescence for Cat D and LAMP-2, a marker for lysosomes. As seen in Figure 5A (upper), normal neurons contain a few lysosomes in the neuronal cell body and Cat D is localized with the lysosomes. However in the MPTP-treated monkey CN sections (Figure 5A lower), we see a drastic increase in the number of lysosomes throughout the neuronal cell body as well as in Cat D immunoreactivity. Quantification reveals an average of four-fold increase in lysosomes (Figure 5B). Given this increase, we next questioned whether increased lysosomal biogenesis could arise from overexpression of Cat D.

### Changes in Cat D and lysosomes due to Cat D overexpression

**Figure 6:**
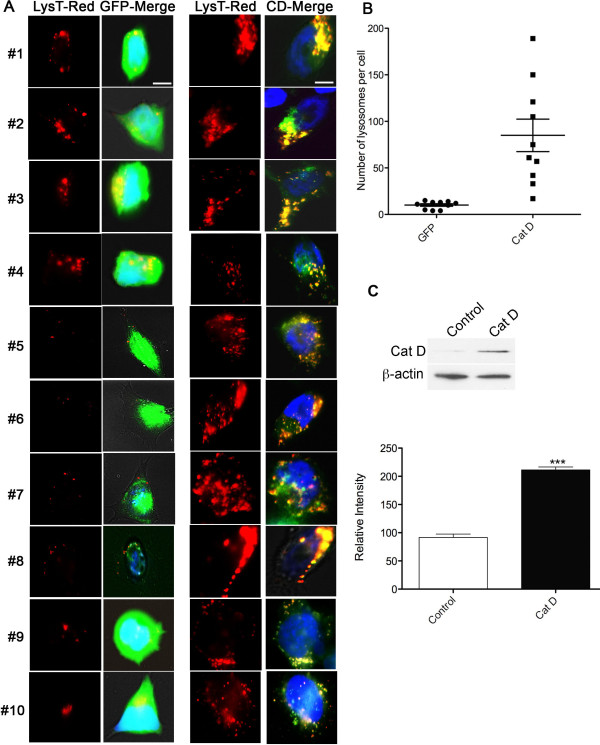
**Overexpression of Cat D in BE-2 cells**. (A) BE-2 neuroblastoma cells were either transfected with GFP (control) or with Cat D-GFP and lysosomes stained with lysotracker red. The GFP expressing BE-2 cells (left panels (LysT-Red, GFP-merge), bar = 5 μm) show few lysosomes localized inside the cell body (see Lysotracker red (LysT-red) panels); whereas, the Cat D-GFP transfected cells (right panels (LysT-Red, CD-merge), bar = 5 μm) show build up of lysosomes (see Lysotracker red (LysT-red) panels). GFP-merge and CD-Merge represent the corresponding merged images with green channel. (B) Quantification of lysosomes was performed on BE-2 cells transfected with either GFP or Cat D (n = 10 cells for each group). Unpaired t-test, p < 0.001 (C) Western blot showing the expression of Cat D (~30 kDa, the mature Cat D heavy chain) in non-transfected (control) and transfected (Cat D) BE-2 cells. β-actin was used as loading control. Lower bar graph is representative of 3 individual experiments. Mean and standard error of the mean shown, significance is indicated by ***p < 0.001.

To investigate the effect of increased Cat D in neurons, we overexpressed human C-terminal GFP-tagged Cat D (Cat D-GFP) or a control GFP (green fluorescent protein) construct in the BE-2 (M17) human neuroblastoma cell line. Using lysotracker red as an indicator of lysosomes in control BE-2 cells, we observed the presence of normal intracellular lysosomes (Figure 6A). In Cat D transfected cells, Cat D-GFP largely co-localizes with lysosomes, and the number of lysosomes was significantly increased relative to controls (Figure 6A, B), consistent with the findings in our *in vivo *model.

### Cat D is present extralysosomaly and extracellularly

**Figure 7:**
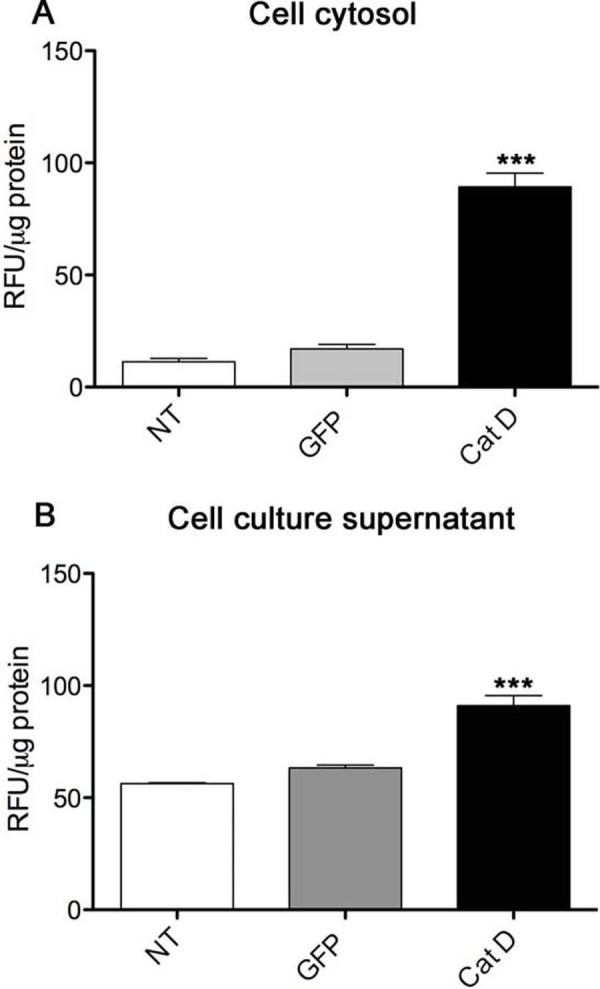
**Cat D is present outside of lysosomes**. Fluorometric assay for cathepsin was performed on BE-2 neuroblastoma cell cytosolic extracts (Figure 6A) and on culture supernatants (Figure 6B) transfected with either GFP or Cat D, or non-transfected (NT). Relative fluorescence unit (RFU) per μg protein is calculated by normalizing the fluorescence signal to the amount of protein present in each sample. The experiments are representative of n = 3. Mean and standard error of the mean shown, significance is indicated by ***p < 0.001.

We next asked if we could detect Cat D enzyme activity in the cytosol and extracellularly. We performed cytosolic fractionation from cellular organelles (Additional file 1) using BE-2 cells, either non-transfected, transfected with GFP alone, or transfected with Cat D-GFP. The Cat D expression as well as activity were measured in cytosolic extracts and in the culture supernatants of the Cat D transfected cells was indeed significantly greater when compared to controls. The active/mature form of Cat D was detected in cytosol (Figure 6C upper panel). Cat D activity assay showed an increase in Cat D activity in the cytosolic fractions as well (Figure 7A, B). Not only can the presence of cytosolic Cat D be deleterious to the cell itself but the presence of extracellular Cat D could be lethal to the surrounding cells. Thus, we subsequently questioned if Cat D expression triggered cell death.

### Overexpression of Cat D leads to caspase activation and apoptosis

**Figure 8:**
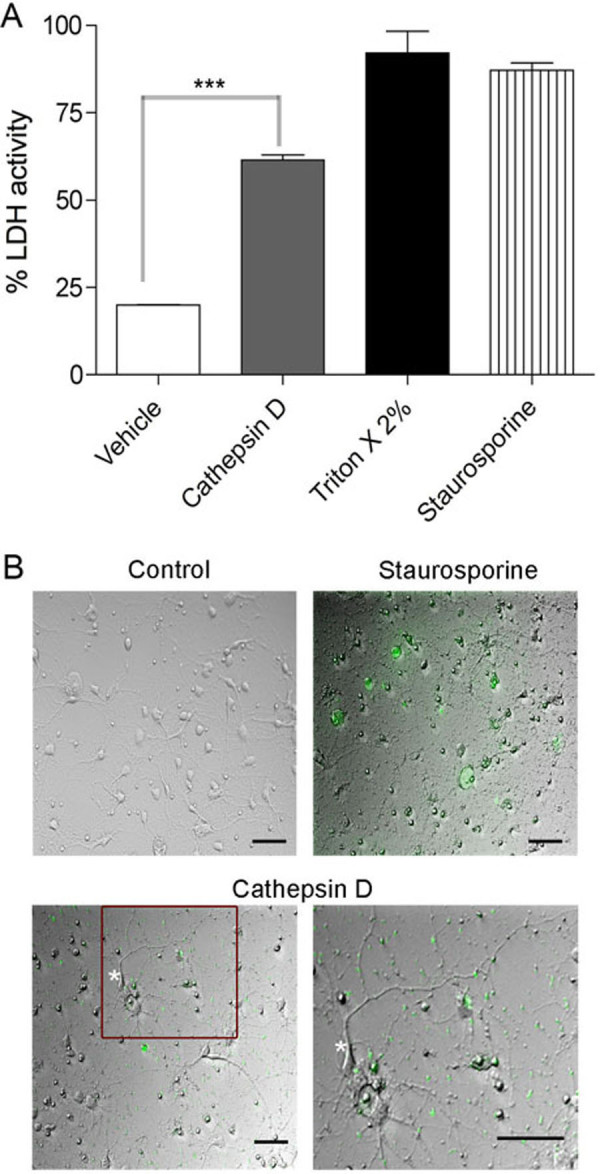
**Cat D overexpression leads to release of lactate dehydrogenase (LDH) and caspase activation**. (A) LDH assay was performed on the rat striatal neurons transfected with either vehicle alone or Cat D. For positive control, 2% Triton X-100 and staurosporine treatments were performed. Cat D overexpression significantly increased the amount of LDH in media. The experiment is representative of n = 4. Mean and standard error of the mean shown, significance is indicated by ***p < 0.001. (B) Live imaging performed on rat striatal neurons. Neurons were transfected with Cat D and stained for CaspACE FITC-VAD-FMK *in situ *marker. The green staining represents the activation of caspase and apoptosis. As seen, compared to control the Cat D transfected neurons show a clear activation in FITC caspase levels, including in neurites (boxed area in lower left panel enlarged in lower right, see processes on neuron marked by asterisk). Staurosporine treated neurons were used as a positive control. Bar = 50 μm.

To investigate whether Cat D overexpression induces pathways leading to neuronal death, we first performed a lactate dehydrogenase (LDH) assay on the culture supernatant, assessing loss of plasma membrane integrity. Figure 8A shows that there is a significant amount of LDH-release caused by overexpressing Cat D in rat striatal neurons when compared to controls. We next performed a fluorescence live imaging assay using the cell-permeable fluorescein isothiocyanate (FITC) conjugate of the caspase inhibitor VAD-FMK. This inhibitor irreversibly binds to the activated caspase, allowing for the *in situ *labeling of cells in which the caspase activation cascade has been initiated. As seen in Figure 8B, when compared to control, the Cat D transfected rat striatal neurons show a greater labeling for caspases, likely due to initiation of apoptosis. Interestingly, caspase activation is also seen along the neurites in Cat D transfected cells (see Figure 8B) indicating neuronal damage.

**Figure 9:**
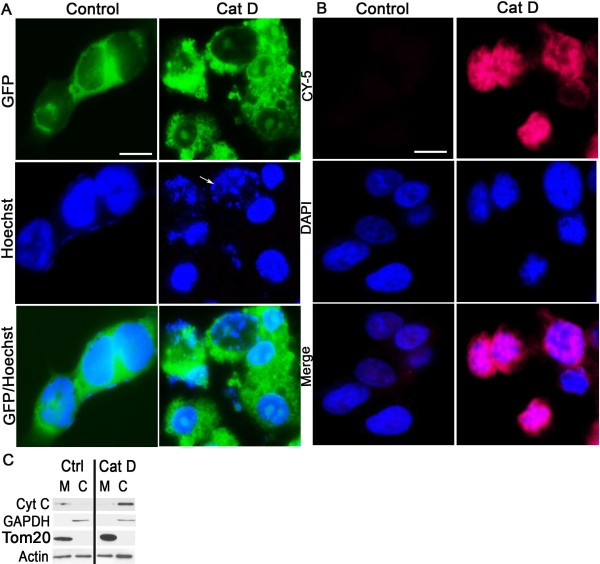
**Cat D overexpression leads to apoptosis**. (A) Hoechst staining was performed on BE-2 neuroblastoma cells transfected with or without Cat D. Cat D overexpression clearly indicates fragmentation of nucleus in Cat-D GFP cells (see white arrow). The experiment is representative of n = 4. Bar = 5 μm (B) *In situ *measurement of apoptosis in Cat D transfected BE-2 neuroblastoma cells. As seen, the CY-5 staining in nucleus can be strongly visualized in Cat D transfected cells but not in control cells indicating the presence of fragmented DNA in Cat D. The experiment is representative of n = 4. Bar = 5 μm (C) Protein extracts from mitochondrial and cytosolic fractions, isolated from Cat D transfected and control cells, were blotted for Cyto-C, GAPDH, Tom20 and β-actin. As can be seen from western blot, there is clear increase in cytosolic presence of Cyto-C in Cat D transfected cells compared to controls, whereas another mitochondrial marker (Tom20) as well as a cytoplasmic marker (GAPDH) remain similar, indicating an activation of apoptotic pathway.

To evaluate the presence of apoptotic cells, Cat D-GFP transfected and control BE-2 cells were stained with Hoechst stain (Figure 9A). The fragmentation of nuclei was clearly observed in Cat D transfected cells when compared to controls. To further strengthen our observation, we performed an *in situ *TUNEL assay. As seen in Figure 9B, fragmented DNA in Cat D transfected cells was clearly labeled. Also, since we found caspase activation, we looked for cytosolic release of Cytochrome C (Cyto-C), an activator for apoptosis. As seen in Figure 9C, there was a clear cytosolic mobilization of Cyto-C in Cat D overexpressing cells confirming its release, which is strongly linked to activation of the apoptotic pathway.

## Discussion

**Figure 10:**
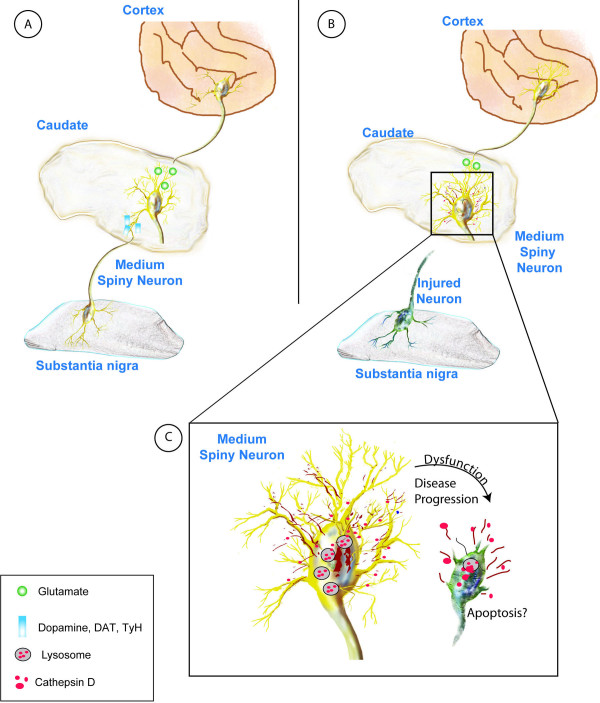
**Schematic representation of disease progression in the caudate**. (A) Depiction of the neuronal pathways involved in normal brain functioning, glutamatergic as well as dopaminergic innervations from cortex and substantia nigra (SN) to the caudate nucleus. (B) MPTP and PD affects the neurons in SN leading to loss of the dopaminergic projections to the caudate, leading to dysregulation of lysosomal pathways, and increasing the number of lysosomes as well as Cat D and extralysosomal Cat D. (C) Slow but persistent damage resulting from upregulation of Cat D expression and leakage leads to neuronal dysfunction and death through apoptosis or other mechanisms.

In human PD a major unmet challenge has been to curb the progression of disease that can affect the higher cognitive functions of the patient. The molecular triggers that induce the progression of disease to areas like the CN and that contribute to associative and executive cognitive functions are currently unknown [2,20,21]. Using a nonhuman primate model of PD, we report for the first time that lysosomal instability and alterations in a lysosomal protease, Cat D, in CN can lead to degeneration and dysfunction of neurons (Figure 10).

In the chronic MPTP model of PD, we first found a significant upregulation of Cat D in the CN. Importantly, we also observed that there is an increased staining for Cat D in neurons, and an increase in the number of lysosomes in neurons in MPTP-induced PD. *In vitro *we found that Cat D overproduction itself led not only to increased lysosomal biogenesis but also led to the occurrence of extralysosomal (including extracellular) Cat D and caspase activation leading to the activation of apoptotic pathway.

While the trigger for increased Cat D production is not known, it has been shown that in order to compensate for the loss of DA in PD, an increased rate of DA turnover takes place in the striatum, especially in the CN. Therefore, these hyperactive neurons are prone to more oxidative stress [4]. Intriguingly, oxidative stress is thought to activate lysosomes and release Cat D into the cytosol in non-neuronal cells [22-26]. Although oxidative stress has been described as a main trigger for the loss of DA neurons in SN pars compacta region in PD [27], its link to PD-related changes in other parts of the brain are less well studied. A complete breakdown of lysosomes and release of high concentrations of lysosomal enzymes can lead to necrosis; whereas partial or selective release of lysosomal proteases could trigger apoptosis [28]. Studies in non-neuronal cells have shown that lysosome membrane permeabilization followed by Cat D release are actually important events in the death cascade and occurs downstream of the Bax-dependent caspase mechanism [29] linking the apoptotic machinery to neuronal loss in our study.

Cell death has been extensively examined in PD, but most of these studies relate to direct DA neuronal loss in the SN pars compacta region, the initiating site of disease [30]. However, we examined the CN, which is not the direct site of injury caused by MPTP but is affected by the progression of disease. Here, we identify the activation of lysosomal pathways as pathogenic in the CN.

Intriguingly, not only was Cat D upregulated in neurons but also was seen to be present in the cytosol and extracellularly, a phenomenon that has not been examined until now in PD or its models. By overexpressing Cat D in human neuroblastoma cells or in rat striatal neurons, we see a similar phenomenon. The deleterious effects of protease leakage in particular, Cat D, into the extracellular environment has been best addressed in breast cancer research where studies indicated that the extracellular matrix was degraded by Cat D released from cancer cells into an acidic microenvironment consequently facilitating tumor invasion [31]. Cancer related studies have also revealed that the presence of cytosolic CD stimulates apoptotic pathways by interacting with members of the apoptotic machinery [32]. Recently, studies also suggest that pro-cat-D secreted by epithelial cancer cells promotes fibroblast outgrowth in a paracrine LRP1-dependent manner in the breast tumor microenvironment [33].

There is precedence for demonstration of extralysosomal Cat D in neurodegeneration. Trimethyltin chloride (TMT) is a potent neurotoxic agent. TMT was shown to upregulate Cat D in neurons both *in vivo *and *in vitro*, and following TMT treatment of rats Cat D is present in the neuronal cytoplasm, and linked to apoptosis [34]. Gaucher's disease is the most common form of lysosomal storage disorders, results in profound neuronal damage, and can be modeled in glucosylceramidase knockout mice. In neurons of such mice Cat D is upregulated and present in the cytosol, and its presence linked to neuronal damage [35]. Alzheimer's disease is the most prevalent neurodegenerative disorder, and aspects can be mimicked in transgenic mice through the expression of mutant forms of amyloid precursor protein in neurons, resulting in intraneuronal amyloid beta accumulation. This again is accompanied by Cat D leakage from lysosomes into the cytoplasm [36].

While our study as well as these support the mechanism that Cat D leakage leads to activation of apoptotic pathway and then cell death, we cannot exclude that Cat D leakage itself can lead to other causes of cellular destruction such as through proteolysis. Lysosomal membrane permeabilization, which includes leakage of Cat D, can result in an apoptotic death as well as cell death that has a subapoptotic or necrotic characteristic [37]. Furthermore there is the possibility that the Cat D leakage is a result of neuronal damage, and not its cause. Although our *in vitro *experiment does not directly address the cause of the leakage phenomenon *in vivo*, the increased Cat D mRNA as well as protein expression definitely is indicative of dysregulated lysosomal pathways, and this could be addressed more effectively in additional animal studies in the future. While cognizant of these caveats, our data provides evidence that leakage of Cat D can be the cue for spread of disease to other regions of the brain in PD.

While others have suggested that increasing expression of Cat D could be used as a therapeutic strategy in PD as it clears toxic α-synuclein aggregates [38], our data clearly indicate that over-production or increased Cat D levels in neurons can lead to neuronal injury, raising caution to this approach, as well as providing pathogenic clues to the progressive neuronal damage in PD.

## Conclusions

In conclusion, our study provides strong evidence that Cat D can be involved in the long-term neuronal damage in PD in regions remote from the site of primary insult, and underscores the need to study brain regions outside the sites of initial damage in neurodegeneration to help understand the basis of disease progression. Therefore, this study provides new avenues for therapeutic interventions that might help to treat or arrest the progression of motor and cognitive disorders commonly seen in human PD patients.

## Materials and methods

### Animals

Adult (6-8 years of age) male rhesus monkeys served as subjects or controls for this study. Monkeys, free from SIV (simian immunodeficiency virus), type D simian retrovirus, and herpes B virus were obtained from Covance (Alice, TX, USA) and Valley Biosystems, Inc. (Sacramento, CA, USA). Animals were kept in a biocontainment facility and housed individually in stainless-steel cages, which permitted olfactory, visual, and auditory contact with other monkeys in the room. Monkeys were paired housed during several hours of the day, which permitted animal interaction and evaluation of social behavior. The room was maintained on a 12/12 hr light/dark cycle (lights on 0600; lights off 1800) and at a temperature of 25 ± 1°C. Monkeys had previously been trained on a BMS task as previously described [39]. The task was designed to test bimanual motor coordination, procedural learning and motivation to work for a preferred food (raisins). Due to the hazardous nature of MPTP, the treated animals (#543, #547 and #549) were kept together in a separate room in the facility. The control animals (#516, #517 and #546) did not receive vehicle injections; they did receive ketamine injections for blood draws. The controls were chosen to be of equivalent age as the experimental group.

### MPTP treatment of nonhuman primates

MPTP hydrochloride (Sigma-Aldrich, St. Louis, MO, USA) was dissolved in sterile saline and given intramuscularly to lightly anesthetized (5 mg/kg ketamine) rhesus monkeys on two consecutive days at an initial dose of 0.4 mg/kg. For clinical rating of Parkinsonism, the Kurlan scale was used in which monkeys were rated daily on weekdays for posture, gait, tremor, general mobility, hand movements, climbing, holding food, eating, balance, gross motor skills, and defense reaction as described [17] in addition to BMS. Animals were tested until recovery from acute effects of MPTP were determined by a stable rating score and were evaluated for the next round of dosing, which was given at doses of 0.3-0.4 mg/kg. The time scale of dosing is indicated in Figure 1, and the overall amount given to each animal is listed in Table 1.

All experiments were performed under the approval of the Environmental Health and Safety Department, the Institutional Biosafety Committee and the Institutional Animal Care and Use Committee of The Scripps Research Institute, following NIH guidelines.

### RNA isolation and Quantitative Real Time PCR

The CN was dissected from the brains, snap frozen, and RNA isolated using Trizol (Invitrogen, Carlsbad, CA, USA). RNA was quantified and determined to be of good purity by spectrophotometric analysis on a NanoDrop 8000 (Thermo Scientific, Wilmington, DE, USA). To check the integrity, RNA samples were run on the Bioanalyzer 2100 (Agilent Technologies, Santa Clara, CA, USA). The RIN values in the range of 7-9 for the RNA samples were indicative of good quality.

Two micrograms of RNA from the CN of control and MPTP monkeys were used for reverse transcription. In a 50 *μ*L reaction, reverse transcription (RT) was carried out using the Superscript kit (Invitrogen) for 1 hr at 42°C, followed by 70°C for 5 min to inactivate the RT reagents. RNase H (New England Biolabs, Beverly, MA, USA) treatment was then performed at 37°C for 20 min. An equal volume of RNase and DNase free water was then added to the reactions.

Real time PCR was performed using gene-specific primers and probes. The primers and probe sequences were designed for rhesus sequences using the Genescript online tool http://www.genescript.com and obtained from Eurogentec (San Diego, CA, USA). The sequences of the primers and probe for 18S and GAPDH controls have been previous reported [40]; whereas, the ones for Cat D were based on the rhesus Cat D sequence (GenBank accession XM_001091601.2): forward primer ACTGCAAACTGCTGGACATC, reverse primer CGTAGTGGATGGCAAATGAG, probe CAGCGACAAGTCCAGCACCTACG. Dilution analysis was carried out using monkey spleen cDNA on all the primers used for the study. The resulting Ct values indicated good linearity over the dilutions, with R^2 ^values ranging from 0.994 to 0.998, and the efficiencies calculated from the slopes were from 95 to 100%.

To carry out quantitative real time PCR, 2 *μ*L of the (1:10 diluted) cDNA was used for assaying the amount of 18S endogenous rRNA; 5 *μ*L (undiluted) each for all other genes. All reactions were performed in duplicates, 12.5 *μ*L of Taqman gene expression master mix (Applied Biosystems, Foster City, CA, USA) was added per 25 *μ*L reaction. The reaction mixture was brought to a final concentration of 5 mM MgCl_2_. Real time PCR was performed in 96-well plate on a StepOnePlus real time PCR system (Applied Biosystems). The delta Ct (dCt) method was performed to determine relative concentrations using the average of the Ct of 18S and GAPDH as the normalizing value. Relative units (2^dCt^) were calculated and used as a measure of mRNA expression. Unpaired student's *t *tests (GraphPad Prism Software, San Diego, CA, USA) were used for statistical analyses.

### Immunohistochemistry and Double Immunofluorescence

Formalin-fixed, paraffin-embedded tissue blocks containing the striatum (caudate nucleus and putamen) were sectioned at 5 μm thickness and picked up on glass slides. For immunohistochemistry, following deparaffinization, antigen retrieval was performed by heating to 95°C in 0.01 M citrate buffer, pH 6.39, for 40 min, then left for 20 min to steep. Sections were blocked in 0.5% casein, followed by the addition of either 1:1000 dilution of an antibody to TyH (Millipore, Billerica, MA, USA), 1:1000 dilution of an antibody to DAT (Millipore) or 1:50 dilution of an antibody to Cat D (Cell Signaling, Denvers, MA, USA) overnight at 4°C. Following washes, signal was detected using the SuperPicture broad spectrum secondary antibody-horseradish peroxidase polymer reagent (Invitrogen) and developed with the 3,3'-Diaminobenzidine (DAB) (Vector Laboratories, Burlingame, CA, USA), followed by a hematoxylin counterstain (Invitrogen) and examined microscopically (Zeiss inverted microscope, Carl Zeiss, NY, USA).

For double immunofluorescence, sections were deparaffinized, antigen retrieval was performed by heating to 95°C in 0.01 M citrate buffer, pH 6.39, for 40 min, then left for 20 min to steep. Sections were blocked in 20% BSA, followed by the addition of a 1:50 dilution of rabbit polyclonal anti-Cat D antibody (Cell Signaling) overnight at 4°C. Following PBS washes, sections were incubated in chicken anti-rabbit Alexa-Flour 488 secondary antibody (Invitrogen) for 1 hr, followed by blocking in 20% BSA and incubation with 1:100 dilution of rat monoclonal anti-LAMP2 antibody (Abcam, Cambridge, MA, USA) overnight at 4°C. Following washes, sections were incubated in goat anti-rat Alexa-Flour 568 secondary antibody (Invitrogen) for 1 hr followed by final washes, mounting in Prolong gold anti-fade with DAPI (Invitrogen). For MAP2 double staining, following staining and detection of Cat D, mouse monoclonal anti-MAP2 (Sternberger Monoclonals, Covance, Princeton, NJ, USA) antibody was used in conjunction with goat-anti-mouse Alexa-Flour 568 secondary antibody (Invitrogen). For microglial double immunofluorescence, the above staining protocol was followed, except using mouse monoclonal anti-Cat D (Abcam) followed by goat-anti-mouse Alexa-Fluor 568 as a secondary antibody, and rabbit polyclonal anti-IBA1 (Wako, Richmond, VA, USA) followed by goat-anti-rabbit Alexa-Fluor 488 secondary antibody (Invitrogen). We interchanged the IBA1 color to red and Cat D to green using the AxioVision REL 4.8 Software (Carl Zeiss, NY, USA) so that the images appear consistent throughout the panel.

### Quantification of lysosomes

Images were taken as *z-stacks *at 63 × for larger magnification. A single neuron was selected from the image, and a final merged image was generated (Cat D/LAMP2). The number of round lysosomes in each plane of the *z-stack *was quantified using a manual counting system recommended by the software. Each plane was scanned for presence of lysosomes, which might be overlayed or hidden in different planes. To prevent duplication, the software generates an identification number for each lysosome. To allow the end-user to accurately determine the presence of a single large vesicle or multiple small ones, double-labeled lysosomes were quantified geometrically as well as densitometrically. Quantification was performed using imaging program, AxioVision REL 4.8 Software (Carl Zeiss, NY, USA). The data were analyzed in Graphpad Prism (GraphPad Software, La Jolla, CA, USA).

### Cell culture

Human neuroblastoma BE-2 (M17) cells (American Type Culture Collection, Manassas, VA, USA) were cultured under sterile conditions in Dulbecco-modified Eagle medium (LONZA BioWhittaker, Portsmouth, NH, USA) supplemented with 10% fetal calf serum, penicillin (100 units/ml), and streptomycin (100 μg/ml) in 5% CO_2 _humidified atmosphere at 37°C.

For isolation of primary rat neurons, embryonic (gestational day 18) rat striatum was purchased from Brain Bits LLC (Springfield, IL, USA). Neuronal cultures were prepared by dissociating the tissue with 0.25% trypsin for 30 min, neutralized with 10% fetal bovine serum, and further dissociating by triturating. The resulting single-cell suspension was centrifuged (1000 rpm/5 min) and was cultured on poly-D-lysine coated plates in Neurobasal media containing 0.5 mM l-glutamine, 50 μg/ml penicillin and streptomycin and supplemented with B27 (Invitrogen). The striatal neurons were grown for 11 days *in vitro *(DIV) and were stained with anti-TyH (Millipore) and anti-DAT (Millipore) to confirm purity.

### Transfections and imaging of rat striatal neurons and BE-2 (M17) cells

After DIV 11 in culture, the rat striatal neurons were transfected with 1 μg human Cat D tagged with GFP at the carboxy-terminus (Origene Technologies, Rockville, MD, USA) or a GFP-only vector (Origene) by NeuroMag according to manufacturer's protocol (OZ Biosciences, Marseille, France). Neurons were cultured for 48 hr after transfection and checked microscopically for GFP expression.

Transfection of BE-2 (M17) cells were done using Fugene 6 (Roche Applied Science, Indianapolis, IN, USA) as per manufacturer's instructions. After transfections, cells were fixed with 4% PFA for 15 min. Double immunoflourescence was performed on the lysotracker treated cells; cells were washed 3 times with 1× PBS for 5 min each wash. Cells were blocked in 10% normal goat serum, followed by the addition of a 1:50 dilution of rabbit polyclonal anti-Cat D antibody (Cell Signaling) overnight at 4°C. Following washes, cells were incubated in goat anti-rabbit Alexa-Flour 488 secondary antibody (Invitrogen) for 1 hr followed by final PBS washes, mounting in Prolong gold anti-fade with DAPI (Invitrogen). Double-labeled lysosomes were quantified as described in this methods section.

### Cathepsin D (Cat D) enzyme activity

Cat D activity was measured in cytosol and in cell culture supernatants of BE-2 (M17) cells transfected with Cat D. Cytosol was prepared by lysing cells in a hypotonic buffer (0.32 M sucrose, 10 mM Pipes pH 7.4, 0.1 M NaCl, 3 mM MgCl_2_, 5 mM EDTA and 0.5% Triton X-100). Cell debris and nuclei were spun down at a low speed, 800 × g for 10 min, and the supernatants were spun at a high speed, 100,000 × g in a TLA100.4 for 1 hr. The supernatants contain the cytosolic contents, and the pellet contains other sub-cellular organelles.

Cat D activity was measured by using a kit containing a fluorogenic peptide substrate peptide, GKPILFFRLK(Dnp)-DR-NH2 labeled with MCA (Abcam, Cambridge, MA, USA). Reactions were initiated by the addition of substrate, and kinetics of substrate hydrolysis was measured using a fluorescent plate reader (Ex 340 nm, Em 460 nm). Data were imported to Graph Pad Prism for analysis and normalization to total protein assayed.

### Lactate dehydrogenase (LDH) assay and CaspACE FITC live-dead assay

LDH-assay was performed as per kit instructions (Cytotoxicity Detection Kit, Roche). Briefly, rat striatal neurons were transfected with Cat D; a vehicle treated was used as a control. As a high control, some neurons were exchanged with assay medium containing 2% Triton X-100, and only assay medium was measured as low control or background control. Some neurons were also treated with 0.5 mM staurosporine (Roche), a positive modulator of cell death. An equal amount of reaction mixture was added to assay medium, and LDH release was measured spectroscopically at 490 nm using a fluorescent plate reader.

For live imaging of apoptotic neurons, CaspACE FITC-VAD-FMK *in situ *marker (Promega, Madison, WI, USA) was used. Briefly, the transfected neurons were incubated with 10 mM of CaspACE FITC-VAD-FMK at 37°C for 30 min. Cells were rinsed in PBS and examined in a fluorescence microscope (Zeiss inverted microscope).

### TUNEL staining

TUNEL staining was performed using the ApopTag Plus Peroxidase *In situ *Apoptosis Detection kit (Millipore) according to the manufacturer's instructions with minor modifications. Briefly, cells were fixed on coverslips with 1% paraformaldehyde, followed by washes and post-fixation for 5 min at -20°C with ethanol:acetic acid (2:1), followed by PBS washes. Samples were oxidized for 5 min with 3% H_2_O_2 _in PBS to reduce endogenous peroxidase activity and then washed with PBS. Slides were next prehybridized in equilibration buffer for 5 min, followed by hybridization with terminal deoxynucleotidyl transferase enzyme for 1 hr at 37°C. The hybridization reaction was terminated by incubation with stop buffer and PBS wash. HRP-conjugated anti-digoxigenin was added next (30 min at RT), followed by PBS wash. The signal was converted to fluorescence using TSA Cy5 kit (Perkin Elmer, MA, USA) according to the manufacturer's protocol. Coverslips were mounted in Prolong gold anti-fade reagent with DAPI (Invitrogen).

### Western blotting

SDS-PAGE electrophoresis was performed using NuPAGE gel system (Invitrogen, Carlsbad, CA) in 4-12% gradient gels under reducing conditions. For western blot analyses, 10 μg of protein extracts were loaded per lane. Nonspecific antibody binding was blocked using 5% nonfat dried milk for 1 hr at room temperature. Immunoblotting was carried out with polyclonal rabbit anti-Cat D (1:1000, Cell Signaling), monoclonal rabbit anti-LAMP1 (1:1000, Cell Signaling), or polyclonal rabbit anti-β-actin (1:5000, Thermo Fisher Scientific); followed by secondary antibody (1:20,000 HRP-conjugated anti-rabbit IgG; Thermo Fisher Scientific). Blots were developed with 1:1 solution of Super Signal West Pico Chemiluminescent Substrate and Luminol/Enhancer (Thermo Fisher Scientific, Rockford, IL, USA).

## Abbreviations

AD: Alzheimer's Disease; BMS: bimanual motor skills; Cat D: cathepsin D; CN: caudate nucleus; CytC: cytochrome C; DA: dopamine; DAB: 3,3'-Diaminobenzidine; DAB: 3,3'-Diaminobenzidine; DAT: dopamine transporter; dCT: delta CT; DIV: days *in vitro; *FITC: fluorescein isothiocyanate; GFP: green fluorescence protein; L-DOPA: L-3,4-dihydroxypheylalanine; LDH: lactate dehydrogenase; MPTP: 1-methyl-4phenyl-1,2,3,6-tetrahydropyridine; NT: non-transfected; PBS: phosphate buffered saline; PD: Parkinson's disease; RFU: relative fluorescence unit; RT: reverse transcription; SN: substantia nigra; TMT: trimethyltin chloride; TyH: tyrosine hydroxylase

## Competing interests

The authors declare that they have no competing interests.

## Authors' contributions

SY carried out the *in vitro *experiments, helped design experiments and wrote the first draft of the manuscript. ADC assisted in the *in vitro *studies. CF performed work with the nonhuman primates. HF performed nonhuman primate work and designed experiments. All authors read and approved the final version of the manuscript.

## Supplementary Material

Additional file 1 **Figure S1**. Click here for file
