# Supplementary figures and images for: Upregulation of cathepsin D in the caudate nucleus of primates with experimental parkinsonism
Source: Mol Neurodegener. 2011 Jul 21;6:52. doi: 10.1186/1750-1326-6-52 (PMC3160400; doi:10.1186/1750-1326-6-52)

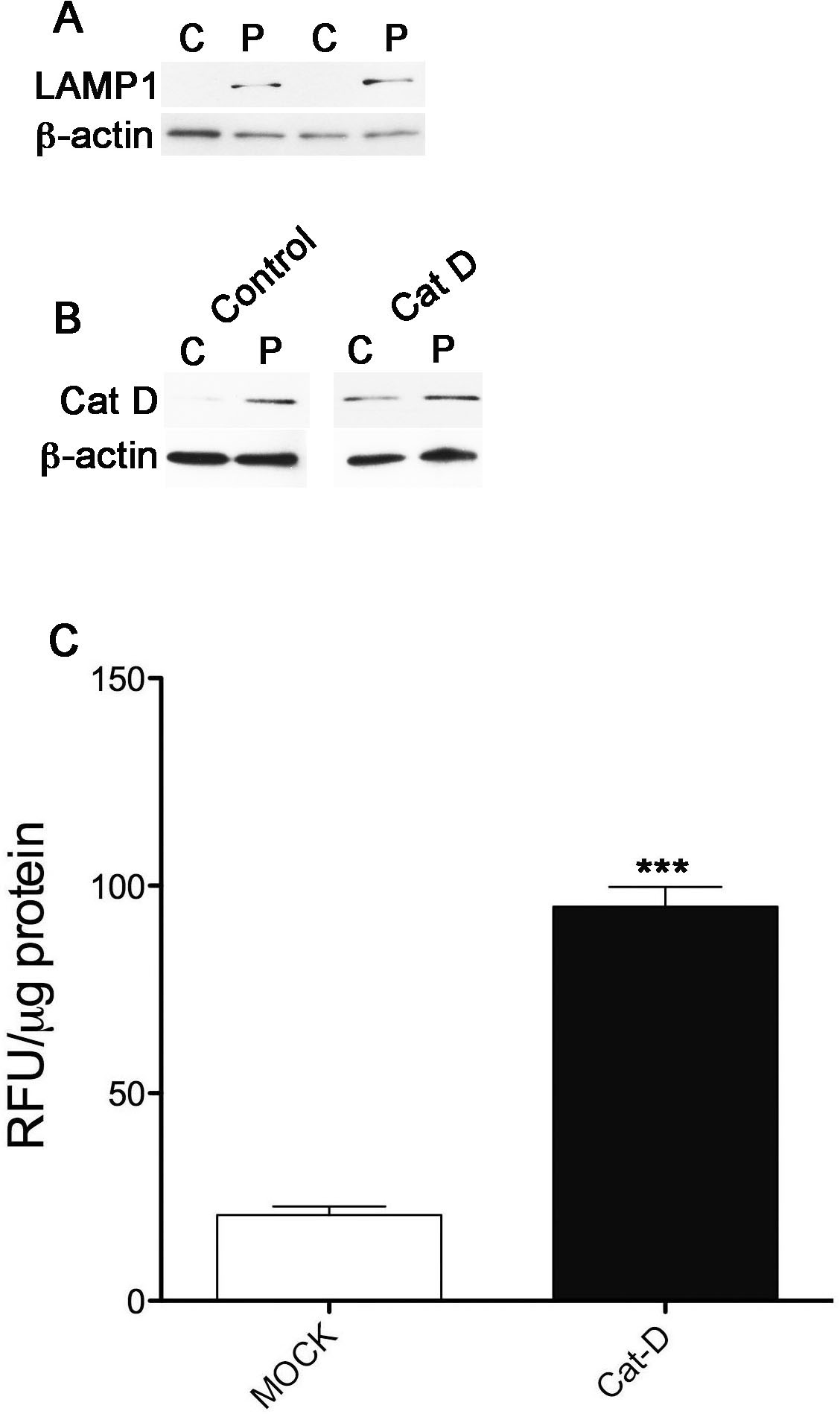

Supplement: Additional file 1 — Figure S1. [file 1750-1326-6-52-S1.JPEG]
